# Onasemnogene abeparvovec gene replacement therapy for the treatment of spinal muscular atrophy: a real-world observational study

**DOI:** 10.1038/s41434-022-00341-6

**Published:** 2022-05-24

**Authors:** Ilaria Bitetti, Valentina Lanzara, Giovanna Margiotta, Antonio Varone

**Affiliations:** 1 Pediatric Neurology, Santobono-Pausilipon Children’s Hospital, Naples, Italy; 2 Department of Pharmacy, Santobono-Pausilipon Children’s Hospital, Naples, Italy

**Keywords:** Neurological disorders, Biomarkers

## Abstract

Spinal muscular atrophy (SMA) is a genetically inherited recessive neuromuscular disease that causes muscular atrophy and weakness. Onasemnogene abeparvovec (formerly AVXS-101, Zolgensma®, Novartis) is a targeted therapy approved to treat patients with SMA in >40 countries worldwide. This study describes the clinical efficacy and tolerability of gene replacement therapy with onasemnogene abeparvovec over a 3-month period in 9 SMA type 1 patients aged 1.7–48 months, with 7 patients on stable nusinersen (i.e., had received all four nusinersen loading doses before inclusion in this study). Liver function (alanine aminotransferase, aspartate aminotransferase, total bilirubin), troponin I, platelet counts, creatinine levels, and motor function (CHOP-INTEND) were monitored. For the seven patients on stable nusinersen, the median baseline CHOP-INTEND score increased significantly during nusinersen treatment (Wilcoxon signed-rank test *p* = 0.018) and at 3 months after switching to onasemnogene abeparvovec (Wilcoxon signed-rank test *p* = 0.0467). We also identified two patients who responded poorly to nusinersen but showed the largest increase in baseline CHOP-INTEND scores at 1 and 3 months after switching, which could suggest that poor responders to nusinersen may respond favorably to onasemnogene abeparvovec. No unknown adverse events occurred. One patient developed moderate/severe thrombocytopenia 1 week after onasemnogene abeparvovec administration that resolved after treatment. Our study suggests the possibility of a change in the dynamic of CHOP-INTEND for patients who respond poorly to nusinersen after switching therapy to onasemnogene abeparvovec. Alternatively, patient age at treatment initiation may impact the response to onasemnogene abeparvovec. Testing in larger patient populations must be undertaken to assess the plausibility of these hypotheses.

## Introduction

Spinal muscular atrophy (SMA) is a genetically inherited recessive neuromuscular disease caused by mutations in the survival motor neuron 1 (*SMN1*) gene [1] located in the 5q13 region on chromosome 5 (reviewed in [2]). SMA is characterized by a loss of motor neurons with subsequent muscular atrophy and weakness, with its severity dependent on the allelic form related to mutations in *SMN1* and the number of *SMN2* copies, an *SMN1* paralog that produces 10–15% of all functional SMN protein [3, 4]. An incidence of approximately 1 in 11,000 live births is reported [5], although this varies globally, with an estimated incidence of 1 in 30,000 to 40,000 live births recently reported in Japan [6]. Implementation of the standard-of-care recommendations for SMA, updated in 2018 [5, 7], and the development and approval of targeted disease-modifying therapies have improved patient survival and modified disease progression.

Onasemnogene abeparvovec (formerly AVXS-101, Zolgensma^®^, Novartis Gene Therapies EU limited, Dublin, Ireland) is an adeno-associated virus (AAV) vector-based gene therapy administered via a single intravenous infusion and designed to deliver a functional copy of the human *SMN* gene across the blood-brain barrier. It is approved to treat patients with SMA in >40 countries worldwide, and is authorized in patients with 5q SMA with a bi-allelic mutation in the *SMN1* gene and a clinical diagnosis of SMA type 1, or up to 3 copies of the *SMN2* gene in the European Union [8], and in pediatric SMA patients aged <2 years with bi-allelic mutations in the *SMN1* gene in the United States [9]. Rapid and early benefits of onasemnogene abeparvovec were demonstrated in symptomatic patients with infantile-onset SMA in the phase 3 STR1VE [10] and STR1VE-EU [11] trials, with evidence of sustained and durable efficacy, as well as a favorable long-term safety profile, shown in the 5-year extension of the phase I trial, START [12]. However, an overview of safety data for onasemnogene abeparvovec identified hepatotoxicity, transient thrombocytopenia, cardiac events, thrombotic microangiopathy, and ganglionopathy as safety risks [13]. Although serious, effective management of these adverse events can be achieved if anticipated and recognized early.

In November 2020, the Neurology Department of the A.O.R.N. Santobono-Pausilipon in Naples, a reference center in the Campania Region for the diagnosis and management of SMA, was identified as a prescribing center for onasemnogene abeparvovec for the treatment, within the first 6 months of life, of patients with a genetic diagnosis of SMA type 1 (bi-allelic mutation in the *SMN1* gene and up to 2 copies of the *SMN2* gene) or with a clinical diagnosis of SMA type 1, in accordance with the law 648 of 23 December 1996. From March 2021, following changes made by AIFA on drug reimbursement [14], it was possible to extend enrollment to children with SMA type 1 who were older than 6 months if they weighed less than 13.5 kg.

This real-world observational study evaluated the clinical efficacy and tolerability of gene replacement therapy with onasemnogene abeparvovec in 9 infants with SMA, 7 of whom were on stable nusinersen. We also describe residual and acquired neuromotor functions before and during nusinersen treatment, and before and after switching to onasemnogene abeparvovec, in this subpopulation of seven patients.

## Methods

The study cohort included patients with SMA treated with onasemnogene abeparvovec between November 2020 and November 2021 and hospitalized in the Department of Neurology, AORN Santobono-Pausilipon, in Naples. To be eligible, patients had to have genetically confirmed 5q SMA with a bi-allelic mutation in the *SMN1* gene and either a clinical diagnosis of SMA type 1 or up to 3 copies of the *SMN2* gene, weighing <13.5 kg, and no contraindications to onasemnogene abeparvovec according to the European Medicines Agency (EMA) technical data sheet [8]. For all patients, the diagnosis of SMA was confirmed with a genetic test identifying the presence of a homozygous deletion of exons 7 and 8 in the *SMN1* gene on chromosome 5q with 95% reliability. No specific exclusion criteria were applied.

All parents provided written informed consent to participate in the study after being informed of the risks and benefits of onasemnogene abeparvovec and therapeutic alternatives, including those of a palliative nature. The local Ethics Committee approved the study.

### Therapeutic protocol

Before administering onasemnogene abeparvovec, preliminary investigations included: blood chemistry tests, including AAV9 antibody dosage, liver function tests (alanine aminotransferase [ALT], aspartate aminotransferase [AST], total bilirubin), creatinine, complete blood count (including hemoglobin and platelet count), troponin I, and instrumental investigations (abdomen ultrasound) [8].

All patients received the standard dose of onasemnogene abeparvovec 1.1 × 10^14^ vg/kg by intravenous infusion over approximately 60 min. Patients also received immunomodulatory therapy with corticosteroids and underwent appropriate motor and respiratory rehabilitation therapy according to the current standard-of-care. In accordance with the EMA technical data sheet [8], prednisolone treatment was started 24 h before gene therapy administration at a standard dose of 1 mg/kg of body weight per day and continued for at least 4 weeks; the steroid dosage was increased only for peaks of liver enzymes >5 x upper limit of normal.

Seven patients had received prior treatment with nusinersen at the recommended dosage of 12 mg (5 ml) per administration, with four loading doses on days 0, 14, 28, and 63, followed by a maintenance dose every 4 months thereafter. Nusinersen was administered by intrathecal bolus injection over 1–3 min by lumbar puncture, after performing an evacuative rachicentesis of a volume of cerebrospinal fluid equal to that injected [15].

Management of patients followed a multidisciplinary perspective, which included neurological evaluation, psychiatric evaluation, and administration of the Children’s Hospital of Philadelphia Infant Test for Neuromuscular Disorders (CHOP-INTEND) functional scale [16], pulmonary examination, speech therapy, and nutritional and pediatric consultations.

### Functional and laboratory assessments

CHOP-INTEND was used to evaluate the motor skills of patients with SMA on stable nusinersen (i.e., patients who had received all four nusinersen loading doses before inclusion in this study) before the start of nusinersen treatment (N-T0) and 1 month after the fourth dose (post-load). Control blood chemistry tests, including blood counts, and renal and hepatic function tests, were also performed periodically to monitor adverse events during nusinersen treatment. CHOP-INTEND also evaluated the motor skills of these patients at baseline (T0) and at months 1 and 3 after intravenous administration of onasemnogene abeparvovec.

All patients who received onasemnogene abeparvovec underwent instrumental and laboratory assessments of adverse reactions, executed at baseline (T0), every 7 days for 1 month, then every 15 days for 2 months, with more frequent monitoring if needed. Monitoring of liver toxicity included measurements of ALT (normal range 8–40 units/L), AST (normal range 5–58 units/L), total bilirubin (normal range 0.10–1.10 mg/dL), including direct (normal range 0.05–0.20 mg/dL) and indirect (normal range 0.10–0.90 mg/dL) bilirubin, and abdominal ultrasound. Other laboratory assessments included troponin I levels (normal range 1–14 ng/L), platelet counts (normal range 140,000–440,000 per mm^3^), and creatinine levels (normal range 0.2–0.45 mg/dL). For all assessments, the normal range reported here reflects the reference range used in our laboratory. General clinical conditions and adverse events were also assessed.

CHOP-INTEND is a validated assessment scale that evaluates patients’ motor skills with early-onset neuromuscular diseases, particularly those with SMA type 1 [16]. It is divided into 16-items, which consider the most clinically significant motor goals for the disease and take into account the limited patient tolerance to acquire and maintain certain postures and the degree of fatigue secondary to respiratory compromise. For each item, scores relating to the two parts are assigned for a maximum total score of 64 points. The best performances are always evaluated.

### Statistical analysis

Categorical variables are reported as number (percentage), mean ± standard deviation (SD), or median (range). For continuous variables, between-group comparisons were performed using the non-parametric Wilcoxon signed-rank test after performing the test of normality (Shapiro–Wilk test) on the entire sample, which rejects the hypothesis of asymmetry. *P*-values *p* < 0.05 were considered statistically significant. Analyses were conducted using IBM^®^ SPSS^®^ software.

## Results

### Patients

The patient cohort consisted of 9 infants with SMA type 1 (6 males, mean age 25 ± 18.8 months, range 1.7–48 months); all carried a homozygous deletion of exons 7 and 8 in the *SMN1* gene on chromosome 5q and had 2 copies of the *SMN2* gene (Table 1). Eight patients had received nusinersen prior to their enrollment in this study (mean age at nusinersen administration 5.3 ± 1.6 months); patient 6 (age 1.7 months) was newly diagnosed and nusinersen-naive.

**Table 1:** Demographic and clinical characteristics of the whole patient population (*n* = 9).

	*n* (%)
Male	6 (66.6)
Female	3 (33.4)
Age of onset (months), mean ± SD (range)	2.7 ± 1.9 (0–5)
Age at diagnosis (months), mean ± SD (range)	3 ± 0.9 (1–7)
Age (months), mean ± SD (range)	25 ± 18.8 (1.7–48)
Nusinersen	8 (88.9)
PEG	2 (22.2)
NIV < 16 h	9 (100)
NIV > 16 h	0 (0)
Tracheostomy	0 (0)
2 copies of the *SMN2* gene	9 (100)

*h* hours, *n* number, *NIV* non-invasive ventilation, *PEG* percutaneous endoscopic gastrostomy, *SD* standard deviation.

All patients retained autonomous respiratory capacity without the need for tracheostomy or permanent ventilation while benefiting from non-invasive ventilation support (NIV) for <16 h/day and were autonomous in feeding, although two patients had a percutaneous endoscopic gastrostomy (PEG) tube due to impaired swallowing function.

### Nusinersen subpopulation

Of the 8 patients treated with nusinersen, patient 1 (age 6 months) received only three of the four loading doses and was excluded from this analysis. For the 7 patients on stable nusinersen, the median baseline CHOP-INTEND score increased significantly by 15 points post-load, from 21 points (range 11 to 32) at N-T0 to 36 points (range 25 to 57) post-load (Wilcoxon signed-rank test *p* = 0.018).

**Fig. 1:**
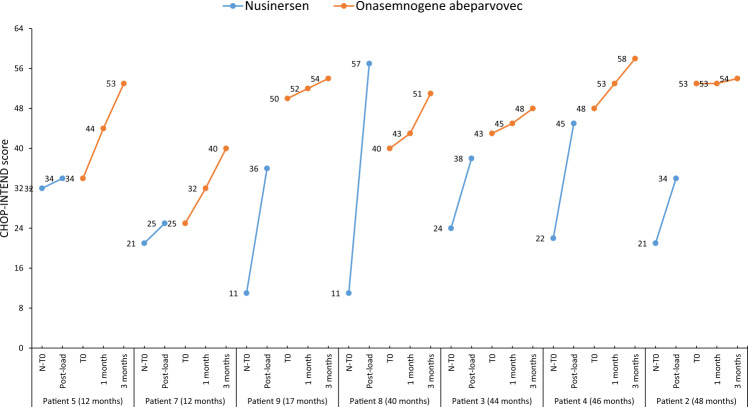
CHOP-INTEND scores for the 7 patients on stable nusinersen who were switched to onasemnogene abeparvovec. CHOP-INTEND scores while on treatment with nusinersen are shown in blue, with assessments taken at the start of nusinersen treatment (N-T0) and 1 month after the fourth dose (post-load). CHOP-INTEND scores during treatment with onasemnogene abeparvovec are shown in orange, with assessments taken at baseline (T0) and at months 1 and 3 after intravenous administration of onasemnogene abeparvovec. The horizontal axis shows patient number and age, with patients listed from youngest to oldest.

When assessed individually, CHOP-INTEND scores increased from N-T0 to post-load by only 2 points in patient 5 (age 12 months) (from 32 to 34 points, respectively) and 4 points in patient 7 (age 12 months) (from 21 to 25 points, respectively); conversely, patient 8 (age 40 months) had an increase of 46 points in CHOP-INTEND score (from 11 points to 57 points, respectively) (Fig. 1).

No adverse events were reported during nusinersen treatment.

### Motor function after onasemnogene abeparvovec administration

CHOP-INTEND for the 7 patients on stable nusinersen increased after onasemnogene abeparvovec administration from a median of 43 points (range 25 to 53) at baseline to 45 points (range 32 to 53) at 1 month and 53 points (range 40 to 58) at 3 months, corresponding to an increase of 2 points (range 0 to 10) at 1 month and 10 points (range 1 to 19) at 3 months, respectively (Wilcoxon signed-rank test *p* = 0.4036 for 1 month and *p* = 0.0467 for 3 months).

When assessed individually, CHOP-INTEND scores for all 7 patients increased over time and were highest at 3 months, with scores >50 recorded in 5 patients at 3 months (Fig. 1). Interestingly, patient 5 and patient 7 (both age 12 months) showed the largest increase in baseline CHOP-INTEND score at months 1 and 3 after switching to onasemnogene abeparvovec (an increase of 10 and 19 points, respectively, for patient 5, and 7 and 15 points, respectively, for patient 7). Conversely, the smallest increase in baseline CHOP-INTEND score was observed in patient 2 (age 48 months) (Fig. 1).

### Drug tolerability and adverse events

Low-grade fever (maximum temperature 38 °C), vomiting, or diarrhea were reported within 7 days of onasemnogene abeparvovec administration by 6, 6, and 3 patients, respectively; all events resolved spontaneously within 3 days of onset (Table 2).

**Table 2:** Adverse events reported in the whole patient population (*n* = 9).

Adverse event	*n* (%)
Fever or low-grade fever	6 (66.7)
Vomiting	6 (66.7)
Diarrhea	3 (33.3)
Thrombocytopenia	5 (55.6)
Moderate/severe	1 (11.1)
Mild	4 (44.4)
Hypertransaminasemia	7 (77.8)
Liver echogenicity changes	1 (11.1)
Increased troponin I	9 (100)

*n* number.

**Fig. 2:**
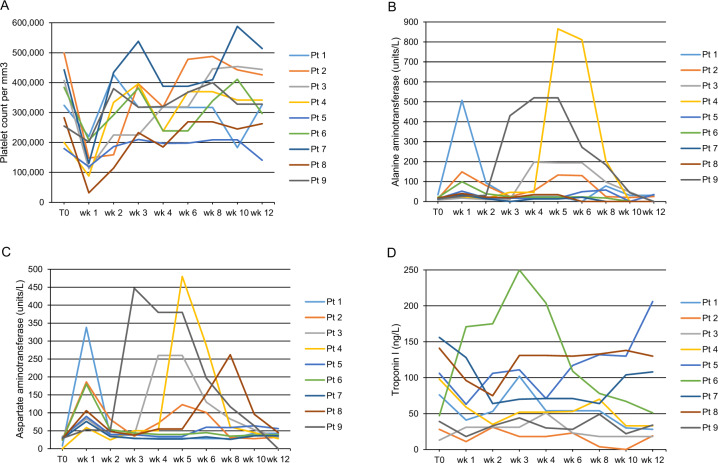
Laboratory assessment outcomes for all patients who received onasemnogene abeparvovec over the 3-month study period, with assessments taken at baseline (T0), every 7 days for 1 month, then every 15 days for 2 months. Levels of platelet counts (**A**), alanine aminotransferase (**B**), aspartate aminotransferase (**C**), and troponin I (**D**) are shown. Each patient is shown using a different line color, which corresponds across all four panels.

One patient developed moderate/severe thrombocytopenia 1 week after onasemnogene abeparvovec administration (platelet count 32,000 per mm^3^) (Fig. 2A). The deficit was treated with a bolus of Ig vein 0.8 mg/kg, and platelet counts normalized within 2 weeks. Mild thrombocytopenia (platelet count 50,000 to 140,000 per mm^3^) was reported in 3 patients at week 1 and 1 patient at week 3. For these patients, platelet counts normalized within 1 week.

Hypertransaminasemia was reported in 7 patients in the weeks following treatment (maximum ALT value 866 units/L, maximum AST value 480 units/L, both in week 5) (Fig. 2B, C). ALT and AST values normalized after modifying the corticosteroid dosage. One patient experienced altered liver parenchymal echogenicity, identified by ultrasound imaging of the abdomen, which regressed within 2 weeks.

All patients had baseline troponin I values (mean 78.2 ± 47.6 ng/L) that were higher than the normal range (1–14 ng/L), with fluctuation of troponin I values observed throughout the 3-month study period (maximum value of 250 ng/L in week 3) (Fig. 2D). No noteworthy anomalies were detected in clinical and instrumental cardiology evaluations.

Levels of creatinine (0.3 ± 0.02 mg/dL) and total bilirubin (0.16 ± 0.12 mg/dL), including direct (0.08 ± 0.03 mg/dL) and indirect (0.09 ± 0.02 mg/dL) bilirubin, were within the normal range. Lung and bulbar function in terms of the need for increased NIV hours or additional ventilator and nutritional support remained unchanged for all patients.

## Discussion

This single-center, real-world, observational study describes the outcomes of 9 patients with SMA type 1 after gene replacement therapy with onasemnogene abeparvovec over a 3-month study period. Notably, the motor skills of 7 patients on stable nusinersen improved significantly at 3 months after onasemnogene abeparvovec, which was well tolerated with only minor side effects reported.

Our results identified a significant clinical improvement in motor performance 3 months after onasemnogene abeparvovec in a small cohort of 7 patients who switched from stable nusinersen. However, the rate of increase of CHOP-INTEND score was greater with nusinersen than onasemnogene abeparvovec. Specifically, the median baseline CHOP-INTEND score increased by 15 points post-load versus 2 and 10 points, respectively, at 1 and 3 months after onasemnogene abeparvovec. In addition, a recent study, which assessed the efficacy of onasemnogene abeparvovec in 21 nusinersen-treated patients, did not demonstrate a change in the rate of increase of CHOP-INTEND scores for the 6 month period before versus after gene replacement therapy (mean score increase of 6.8 points versus 6.6 points, respectively), although improvement in motor function was observed after switching [17]. Therefore, the benefit of switching patients from nusinersen to onasemnogene abeparvovec remains to be definitively determined.

Nonetheless, it is notable that the two patients who responded poorly to nusinersen in our study (i.e., patients 5 and 7) showed the largest increase in baseline CHOP-INTEND scores at 1 and 3 months after switching. This change in the dynamic of CHOP-INTEND could suggest that patients who respond poorly to nusinersen may respond more favorably to onasemnogene abeparvovec, although the dynamic of CHOP-INTEND evolution is not linear in general [11]. Alternatively, patient age at treatment initiation may impact the response to onasemnogene abeparvovec, with the largest increase in baseline CHOP-INTEND scores observed in the youngest patients (patients 5 and 7, both age 12 months) and the smallest increase observed in the oldest patient (patient 2, age 48 months). However, testing in larger patient populations must be undertaken to assess the plausibility of these hypotheses.

Early detection of SMA via newborn screening, alongside pre-symptomatic treatment, improves outcomes in children with genetically proven SMA [18]. Indeed, numerous published preclinical and clinical data suggest that early treatment is critical to modulate the rapid and progressive degeneration observed in SMA, especially in patients with type 1 [10, 19, 20]. There is also strong evidence that irreversible motor neuron loss in individuals with SMA type 1 begins in the perinatal period, with severe denervation in the first 3 months after birth and the loss of more than 90% of motor units by 6 months of age [21].

Of note, preliminary results, as of the 11 June 2021, of phase 3 SPR1NT clinical trial (NCT03505099) demonstrated significant therapeutic benefits of onasemnogene abeparvovec in pre-symptomatic SMA patients aged ≤6 weeks with 2 or 3 copies of *SMN2*, with some patients achieving gross motor milestones similar to non-SMA infants [22, 23]. Hence, pre-symptomatic treatment with onasemnogene abeparvovec could support neuromotor development in an SMA infant to be broadly similar to that of a healthy child, effectively changing the natural history of the disease [22, 23].

Another factor to consider is the price of onasemnogene abeparvovec, which costs an additional 2,000,000 euros on top of a loading dose of 400,000 euros for nusinersen. However, a recent study calculated that, considering a model-predicted lifespan expected survival was 37.20 years of life for onasemnogene abeparvovec and 9.68 life years for nusinersen, the mean lifetime cost per patient was US$4.2–6.6 million for onasemnogene abeparvovec and US$6.3 million for nusinersen, indicating that onasemnogene abeparvovec was affordable with prices of ≤US$5 million [24].

In our patient cohort, onasemnogene abeparvovec was well tolerated with only minor side effects (low-grade fever or fever, diarrhea, vomiting) reported within 7 days of administration, which resolved spontaneously. Notably, no unknown side effects were observed. Despite baseline troponin I values above the normal range, with transient increases observed after treatment, no noteworthy clinical abnormalities were identified from cardiac assessments. Only 1 patient required Ig vein bolus for thrombocytopenia.

Treatment with onasemnogene abeparvovec is associated with an increased risk of hepatotoxicity, which should be mitigated through appropriate monitoring and intervention [25, 26]. Hepatotoxicity typically presents as non-cholestatic (i.e., as increases in serum aminotransferase concentrations) and most often occurs at 1 week and 1 month after treatment [25]. In our study, increases in AST and ALT above the normal range were primarily seen in week 1, although the largest increases were recorded in week 5. In all cases, elevated transaminases were successfully managed with corticosteroids.

Our findings also suggest that hepatic safety might be a more serious concern in patients previously treated with nusinersen. In our sample, this could be due to patient age at the time of treatment with onasemnogene abeparvovec or in part to previous treatment. Indeed, the experience of clinical studies suggests that the immunogenicity of the AAV vector is, to some extent, dose-dependent [27]. Since the dose is proportional to body weight, the total viral vector load was therefore significantly higher in older children. On the other hand, host-dependent factors could trigger and influence the extent of the immune response, including pre-existing immunity, concomitant infections, age, or genetic background.

Although primarily in line with recent scientific literature and therefore potentially indicative of the acceptable therapeutic standard achieved for onasemnogene abeparvovec, the results of this single-center study are limited by the low patient number and short observation period, which do not allow structured statistical analysis of the data. In addition, as 8 of the 9 patients had previously received nusinersen, it was not possible to determine the isolated impact of onasemnogene abeparvovec on the clinical course of the disease. Furthermore, a significant positive impact on SMA patient survival has been achieved over the last decade following optimization of standard-of-care respiratory support and adequate nutritional management, conditions that could influence the acquisition of motor function milestones.

For the first time in the history of SMA, disease-modifying therapies, such as the modification of splicing and gene therapy, combined with novel diagnostic approaches based on the use of newborn screening alongside pre-symptomatic treatment [18, 19, 22, 23, 28], make it possible to change the natural clinical course of this disease. Despite limitations related to the small patient number and short observation period, results from this single-center study align with previously reported clinical and observational studies and further support the efficacy and safety of onasemnogene abeparvovec in treating patients with SMA type 1. Our study also suggests the possibility of a change in the dynamic of CHOP-INTEND after switching to onasemnogene abeparvovec in patients who respond poorly to nusinersen or that patient age at treatment initiation may impact the response to onasemnogene abeparvovec. However, the plausibility of these hypotheses must be tested in larger patient populations. With other therapeutic approaches in advanced stages of clinical development, including drug combinations [29, 30], a broader spectrum of drug treatment options for SMA is expected in the future, as well as an increase in the complexity of care. Ultimately, longer-term studies in patients with SMA, which allow for higher patient numbers and more extended observation periods, should be implemented to confirm the clinical benefit of onasemnogene abeparvovec on the natural history of the disease.

## Data Availability

The data generated or analyzed during this study can be found within the published article.
